# Case Report: From metabolic normalization to incidental type A aortic dissection in immune checkpoint inhibitor–associated aortitis

**DOI:** 10.3389/fonc.2026.1755873

**Published:** 2026-03-13

**Authors:** Pablo Freijido Alvarez, Luis Angel Leon Mateos, Nerea Gonzalez Garcia, Jorge Garcia Gonzalez, Emilio Huelga Zapico, Miguel Garrido Pumar, Rafael Lopez Lopez

**Affiliations:** 1 Medical Oncology Department, University Clinical Hospital of Santiago de Compostela, Santiago de Compostela, Spain; 2 Oncomet Translational Oncology Group (ONCOMET), Instituto de Investigación Sanitaria de Santiago de Compostela (IDIS), Santiago de Compostela, Spain; 3 Universidade de Santiago de Compostela (USC), Santiago de Compostela, Spain; 4 Medical Oncology Department, Hospital El Bierzo, Ponferrada, Spain; 5 CIBERONC – Centro de Investigación Biomédica en Red de Cáncer (CIBERONC), Instituto de Salud Carlos III (ISCIII), Madrid, Spain; 6 Nuclear Medicine Department, University Clinical Hospital of Santiago de Compostela, Santiago de Compostela, Spain; 7 Radiology Department, University Clinical Hospital of Santiago de Compostela, Santiago de Compostela, Spain

**Keywords:** aortic dissection, aortitis, case report, immune checkpoint inhibitors, immune-related adverse events, large-vessel vasculitis, lung adenocarcinoma

## Abstract

Immune checkpoint inhibitors can precipitate large-vessel vasculitis. It remains unknown whether metabolic remission on 18F-fluorodeoxyglucose positron emission tomography/computed tomography (18F-FDG PET/CT) reliably indicates long-term structural stability or absence of later complications. A 58-year-old man with *KRAS-G12C*–mutated stage IVB lung adenocarcinoma initiated first-line treatment with carboplatin + pemetrexed + pembrolizumab. After the fourth cycle he developed persistent fever with normal procalcitonin and negative cultures. Contrast-enhanced computed tomography showed concentric thickening of the aorta and major branches; 18F-FDG PET/CT demonstrated increased inflammatory uptake consistent with large-vessel vasculitis. Testing for autoimmune and infectious etiologies yielded no diagnostic findings. Given the strong clinicoradiologic agreement and the unfavorable risk–benefit profile of deep arterial biopsy, histologic confirmation was not pursued. Intravenous methylprednisolone led to rapid defervescence and biochemical improvement. On follow-up, 18F-FDG PET/CT demonstrated complete metabolic normalization. Subsequent surveillance imaging incidentally identified an asymptomatic Stanford type A aortic dissection. In the absence of indications for elective repair (diameter below surgical thresholds, no rapid expansion, malperfusion, or significant regurgitation) and after discussion within the multidisciplinary Heart Team, management consisted of structured imaging surveillance and optimal medical therapy. Thereafter, he initiated adagrasib, achieving a durable partial response. This case illustrates discordance between metabolic quiescence and later structural damage in immune checkpoint inhibitor-associated aortitis. This supports long-term structural surveillance, as 18F-FDG PET/CT normalization does not guarantee structural safety.

## 1 Introduction

Immune checkpoint inhibitors (ICIs) can precipitate large−vessel vasculitis/aortitis, yet the natural history of structural disease after apparent inflammatory remission remains poorly defined. Current surveillance practices are largely extrapolated from non−ICI aortitis, and direct evidence linking ICI−aortitis to subsequent aortic dissection remains scant. Reports of Stanford type A dissection occurring months after 18F-fluorodeoxyglucose positron emission tomography/computed tomography (18F-FDG PET/CT) normalization are exceedingly rare; to our knowledge, detailed case−level descriptions of this sequence are virtually absent. We present a case of dissection occurring after apparent aortitis metabolic resolution. The case addresses a critical evidence gap by underscoring that 18F-FDG PET/CT normalization does not equate to structural safety, and it highlights the need for structured, long−term imaging surveillance and individualized cardiovascular management in ICI−associated aortitis.

## 2 Case description

We describe a 58-year-old man with hypertension and dyslipidemia. He was a former smoker (20 pack-years; quit 20 years earlier) and reported weekend alcohol consumption of approximately 2 units. He had no known drug allergies.

In May 2022, he was diagnosed with lung adenocarcinoma, staged cT2a cN3c M1c (stage IVB) based on nodal (left hilar and mediastinal lymph nodes) and central nervous system involvement (a right temporal lobe lesion of 37×30 mm, a premotor frontal cortex lesion of 6.5 mm and a right parieto-occipital cortex lesion of 4.5 mm). Programmed death-ligand 1 (PD-L1) expression by tumor proportion score was negative. Targeted next-generation sequencing of 49 genes (including *EGFR, KRAS, ALK, ROS1, RET*, and *NTRK*) identified *KRAS G12C* mutation (variant allele frequency of 0.203).

Initial management of the brain metastases consisted of dexamethasone (12 mg daily with a planned taper) to control vasogenic edema. On follow-up magnetic resonance imaging, the two subcentimeter lesions had nearly resolved and the dominant right temporal mass had decreased in size. In June 2022, the latter was removed via right temporal craniotomy, with histopathology confirming metastatic lung adenocarcinoma. Given the near-complete radiologic regression of the remaining lesions, stereotactic radiosurgery was deferred.

In July 2022, first-line chemoimmunotherapy was initiated with carboplatin (AUC 5), pemetrexed (500 mg/m²), and pembrolizumab (200 mg IV every 3 weeks), with sequential thoracic radiotherapy to be considered contingent on response. From the first cycle he experienced grade 1 renal dysfunction with a creatinine peak of 0.91 mg/dl (baseline creatinine was 0.7 mg/dl) and a grade 1 cutaneous rash, together with grade 2 anemia. After four cycles he had radiographic stable disease.

In October 2022, on day +14 of cycle 4, he was admitted with febrile neutropenia of unclear source and grade 3 anemia. He improved clinically on empiric meropenem (1 g IV every 8 hours) and supportive care. He was discharged early on oral step-down therapy with levofloxacin 500 mg once daily and cefixime 400 mg once daily.

Five days later he returned with persistent fever of 39 °C, accompanied by marked asthenia, nausea, profuse sweating, hypotension, and jaw discomfort. Laboratory testing showed persistent normocytic, normochromic anemia (hemoglobin 8.2 g/dL), thrombocytosis, worsening renal function (creatinine 1.8 mg/dl), a C-reactive protein (CRP) of 43 mg/L with normal procalcitonin, and an erythrocyte sedimentation rate (ESR) of 55 mm/h.

Empiric broad-spectrum therapy was reinitiated with meropenem (1 g IV every 8 hours) plus linezolid (600 mg IV every 12 hours). Blood and sputum cultures (including induced sputum), polymerase chain reaction testing for respiratory viruses, and serologic testing for atypical bacteria were all negative. A peripheral blood smear supported a hyporegenerative anemia, most consistent with a deficiency state.

## 3 Diagnostic assessment, therapeutic intervention, follow-up and outcomes

During the second admission in October 2022 the clinical course was unfavorable, with persistent febrile spikes and elevated acute-phase reactants, prompting additional diagnostic evaluation.

**Figure 1:**
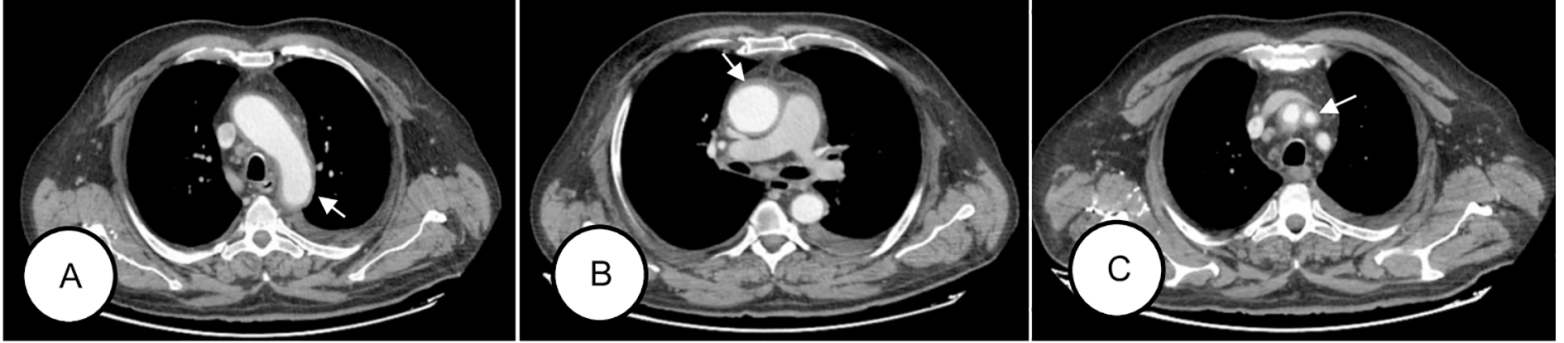
Anatomical extent of vasculitis (CT scan, November 2022, from left to right). **(A)** Involvement of the aortic arch. **(B)** Involvement of the ascending and descending thoracic aorta. **(C)** Involvement of the brachiocephalic trunk, right subclavian artery, origin of the left subclavian artery, and proximal segment of the right common carotid artery. GE Revolution 256-slice CT scanner of the chest, abdomen, and pelvis with biphasic arterial and venous injection of Ultravist iodinated contrast (300 mg I/ml) at a flow rate of 3 ml/s in an antecubital vein was used in the oncological follow-up study where aortic dissection was diagnosed.

Contrast-enhanced computed tomography (CT) revealed concentric mural thickening of the aorta along its entire course and of major branches (the brachiocephalic trunk, both subclavian arteries, and the proximal right common carotid) accompanied by mediastinal fat stranding and new pleural and pericardial effusions (**Figure 1**). 18F-FDG PET/CT demonstrated findings compatible with large-vessel vasculitis (Meller visual scale: grade 3 in the aorta; grade 2 in the supra-aortic trunks, subclavian arteries, and carotid origins) (1). Transthoracic echocardiography showed dilation of the aortic root, whereas temporal-artery duplex revealed no inflammatory changes.

Serologic testing for autoimmunity, including rheumatoid factor (RF), anti–cyclic citrullinated peptide antibodies (anti-CCP), antinuclear antibodies (ANA), antineutrophil cytoplasmic antibodies (ANCA), extractable nuclear antigen panel (ENA), and anticardiolipin antibodies were unremarkable. Serologic testing for *Treponema pallidum* and mycobacterial studies were negative. In view of the compelling clinicoradiologic concordance for large-vessel vasculitis and the limited diagnostic yield and nontrivial risks of arterial wall biopsy of the aorta or supra-aortic trunks (e.g., hemorrhage, dissection, neurovascular injury), histologic confirmation was not pursued, as it was unlikely to alter immediate management.

**Figure 2:**
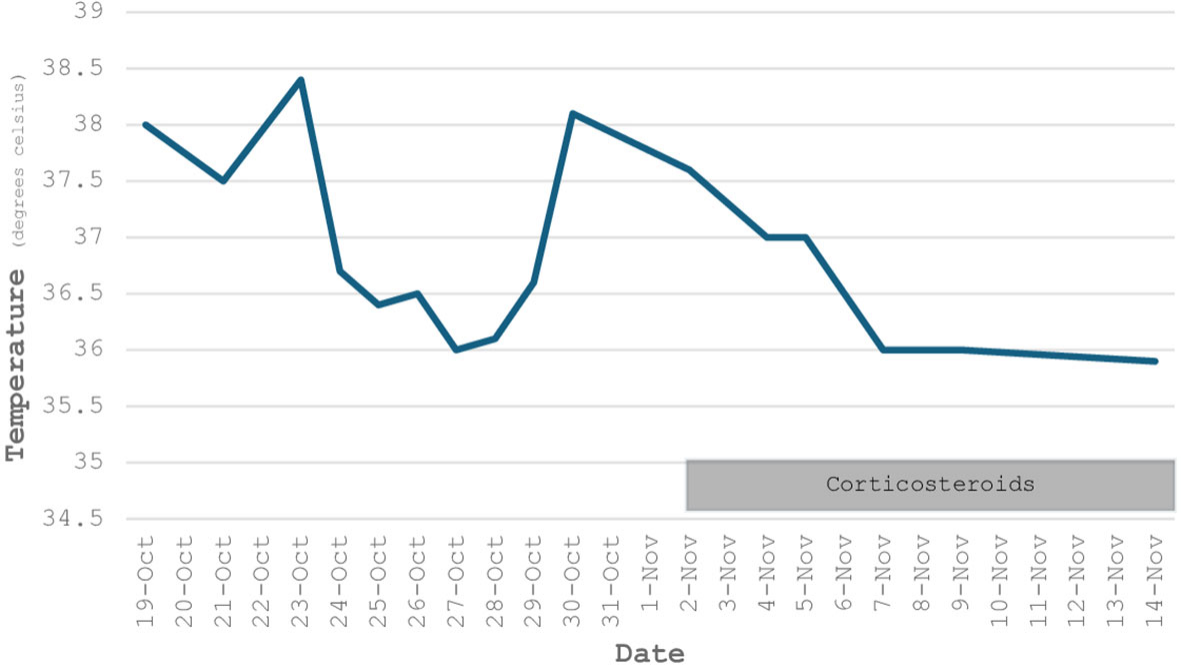
Temperature trend over time. Fever persisted with a protracted course despite antibiotic therapy, in contrast to a rapid defervescence after initiation of methylprednisolone.

Following these findings, intravenous methylprednisolone at 1 mg/kg/day was initiated. This was followed by a rapid clinical improvement with defervescence (**Figure 2**), attenuation of asthenia, rising hemoglobin levels, recovery of renal function, and a marked decline in inflammatory markers.

With a diagnosis of immune-mediated vasculitis and after initial clinical improvement, he was discharged on oral prednisone at 0.75 mg/kg/day with a tapering regimen. Bone protection was instituted with alendronate 70 mg orally once weekly and calcium–cholecalciferol 500 mg/1, 000 IU orally once daily, given underlying osteopenia and the anticipated prolonged corticosteroid course. In addition, Pneumocystis jirovecii prophylaxis was initiated with trimethoprim–sulfamethoxazole 160/800 mg orally, one tablet on Monday, Wednesday, and Friday. Follow-up was coordinated by rheumatology and medical oncology outpatient clinics. The steroid taper was well tolerated, with no recurrence of large-vessel symptoms and no significant adverse effects.

In November 2022, active oncologic therapy was resumed. As disease remained confined to the thorax, local therapy was reconsidered, and concurrent thoracic radiotherapy was initiated with intravenous cisplatin 75 mg/m² and pemetrexed 500 mg/m² administered every three weeks. Treatment was discontinued after the third cycle because of worsening renal function (creatinine 1.9 mg/dl). On assessment in February 2023, a complete response in the central nervous system was maintained with stable thoracic disease. Maintenance pembrolizumab or pemetrexed was not pursued owing to prior toxicity.

**Figure 3:**
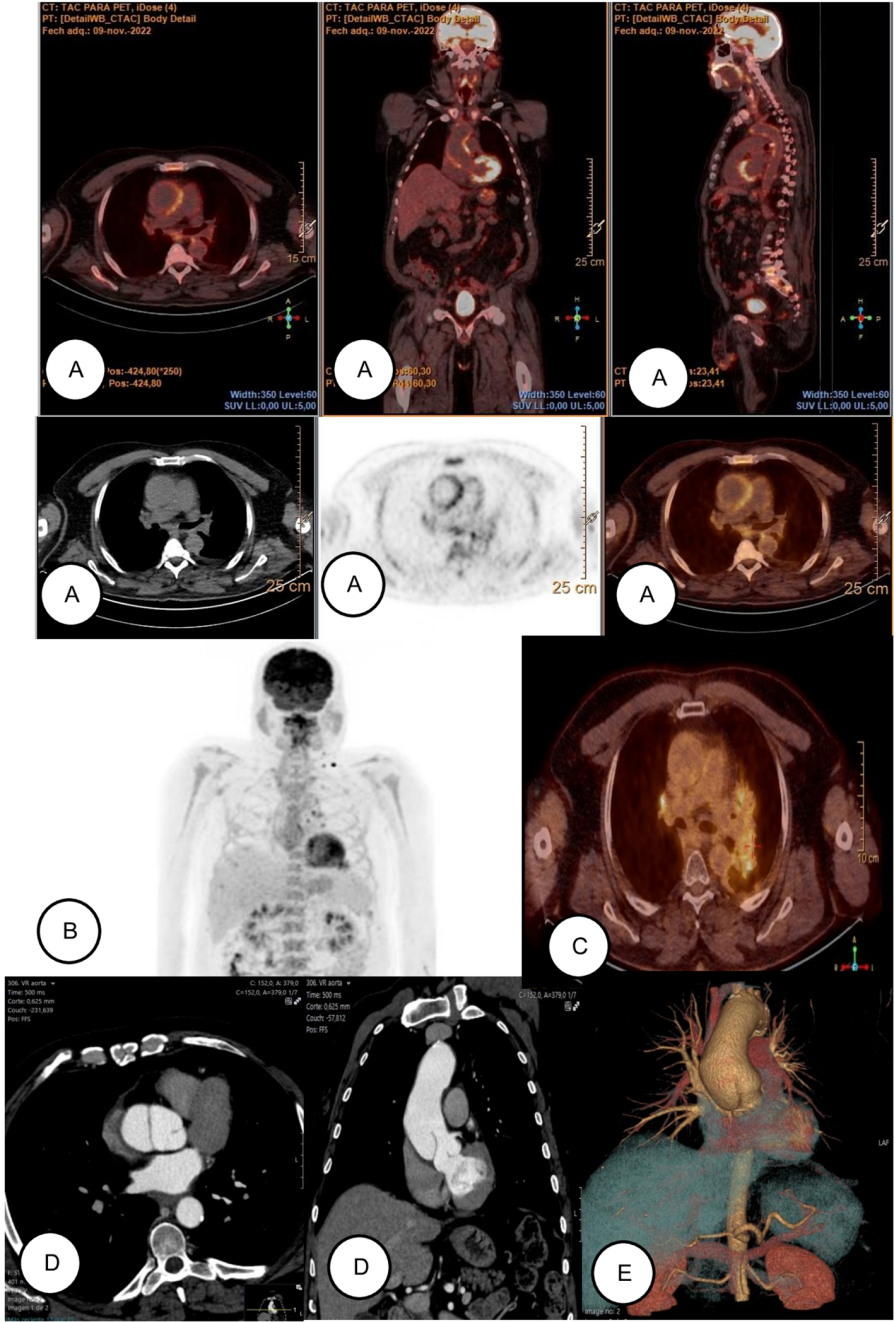
Radiological evolution of vasculitis (images are displayed sequentially from top to bottom and from left to right). **(A)** November 2022: PET/CT performed at diagnosis, demonstrating high-intensity increased metabolic activity (greater than hepatic uptake) in the thoracic aorta. Imaging was performed using a digital PET/CT scanner (Philips Vereos, Philips Healthcare). Image reconstruction was carried out using a three-dimensional ordered-subset expectation maximization (3D OSEM) algorithm. The administered 18F-FDG dose was 3.5 MBq/kg, corresponding to a total injected activity of 356.3 MBq. Image acquisition started 60 minutes after radiotracer injection. Blood glucose level at the time of injection was 96 mg/dL. Semi-quantitative analysis was performed using standardized uptake values (SUV). SUV was calculated according to the standard formula as the ratio of tissue radioactivity concentration to injected dose normalized by patient body weight. The maximum standardized uptake value (SUVmax) measured at the affected aortic wall was 4.89. The aorta-to-blood pool SUV ratio was 3.0, with a mean blood pool SUV of 1.6. The aorta-to-liver SUV ratio was 1.87, based on a mean hepatic SUV of 2.7. **(B)** November 2022: coronal maximum intensity projection (MIP) reconstruction demonstrating increased metabolic activity along the aortic wall. **(C)** May 2023: Follow-up PET/CT with imaging resolution, showing normalization of thoracic aortic metabolism. **(D)** October 2023: axial and coronal images where intimal flap and aneurysmal dilation are observed in the proximal ascending thoracic aorta. **(E)** October 2023: Volume rendering 3D reconstruction.

Subsequently, he transitioned to clinical surveillance. On 18F-FDG PET/CT in July 2023, vascular uptake had normalized with resolution of the previously described pathologic hypermetabolism (**Figure 3**). In September 2023, he was hospitalized for neurologic symptoms and was ultimately found to have intracranial progression. He received whole-brain radiotherapy (20 Gy in five fractions) and a corticosteroid course with clinical improvement. Incidentally, the October 2023 surveillance contrast-enhanced CT demonstrated findings compatible with a Stanford type A aortic dissection, while he remained asymptomatic in this regard (**Figure 3**). In this case, indications for elective surgery were absent (subthreshold aortic diameter, no rapid expansion, malperfusion, or significant regurgitation). After discussion within the multidisciplinary Heart Team and in view of the patient’s oncologic context, the anticipated operative risk was considered to outweigh the potential benefit of elective repair. Consequently, close imaging surveillance with optimal medical therapy was selected. Serial imaging was scheduled at predefined intervals, with earlier reassessment planned in the event of accelerated aortic growth, new symptoms, or other concerning morphologic changes.

In May 2024, the patient enrolled in the KRYSTAL-21 clinical trial and initiated adagrasib, administered orally twice daily with food. At the first assessment in July 2024, a partial response was documented, and this was maintained at the October 2024 evaluation. Adverse events were limited to grade 1 fatigue. In November 2024, oligoprogression was detected in the left fourth rib; given the absence of related symptoms and sustained disease control at other sites, adagrasib was continued, and the disease has remained stable to date (a chronological summary is provided in **Table 1**).

**Table 1:** Chronological summary of clinical events, imaging findings, and therapeutic interventions throughout the patient’s disease course.

Date	Clinical event	Key investigations	Management & outcome
Jul 2022	First-line chemoimmunotherapy (carboplatin AUC 5 + pemetrexed 500 mg/m² + pembrolizumab). Early adverse events: grade 1 renal, grade 1 rash, grade 2 anemia.	—	After 4 cycles: radiologic stable disease.
Oct 2022 (day +14, C4)	First hospitalization: febrile neutropenia (unclear source) with grade 3 anemia.	—	Improved on meropenem IV; early discharge on oral levofloxacin + cefixime.
Oct 2022 (+5 days)	Second hospitalization: persistent fever 39 °C with systemic symptoms.	Labs: anemia, thrombocytosis, ↑CRP, normal procalcitonin, ↑ESR. Microbiology: cultures/polymerase chain reaction/serologies negative.	Empiric meropenem + linezolid IV; unfavorable course → extended work-up.
Late Oct 2022	Large-vessel vasculitis suspected.	CT: circumferential aortic wall thickening. 18F-FDG PET/CT: aortic and supra-aortic inflammatory uptake. Echo: aortic root dilation. Temporal-artery ultrasound: no inflammation. Autoimmune/infectious screen: negative.	No arterial biopsy (strong clinicoradiologic concordance; low yield and non-trivial risk).
Late Oct 2022	Immunosuppression and response.	—	Methylprednisolone 1 mg/kg/day IV → rapid defervescence, symptomatic and biochemical improvement.
Jul 2023	Vascular follow-up imaging.	18F-FDG PET/CT: normalization of vascular uptake (**Figure. 3**).	—
Oct 2023	Incidental vascular finding.	Surveillance CT: compatible with Stanford type A aortic dissection.	Asymptomatic; continued surveillance.
May–Nov 2024	Trial therapy.	—	KRYSTAL-21 trial: adagrasib with partial response; Nov 2024 oligoprogression at left 4th rib → adagrasib continued, disease stable to date.

## 4 Discussion

This case underscores an unexpected sequence in ICI-associated aortitis: complete metabolic quiescence on follow-up 18F-FDG PET/CT imaging followed months later by an incidental, asymptomatic Stanford type A dissection. This dissociation between inflammatory “silence” and later structural damage is seldom documented and has direct implications for practice.

Immune checkpoint inhibitors (ICIs) have transformed the therapeutic landscape of oncology. By blocking inhibitory checkpoints on T cells, these agents enhance antitumor immunity (2). Between 1% and 10% of treated patients experience rheumatologic immune-related adverse events (irAEs), among which arthritis is the most common (3). Less frequently, ICIs precipitate vasculitis that may be organ-limited or systemic (4). These toxicities share features with classic autoimmune diseases, although formal classification criteria are not always fulfilled. Proposed mechanisms involve T-cell activation and cytokine networks (including TNF-α, IL-6, and IL-17), which supports the selective use of biologic therapies in refractory cases.

In a systematic review of 20 cases of ICI-associated vasculitis, the median interval from treatment initiation to vasculitis onset was approximately three months, similar to the interval observed in our patient (5). Most reported cases involved large or medium-vessel disease. Only minor differences have been described between ICI-associated and idiopathic vasculitis with respect to clinical presentation, laboratory findings, and histopathology (6). For example, idiopathic giant cell arteritis exhibits a microenvironment characterized by reduced expression of the inhibitory ligand PD-L1 and an expansion of PD-1–positive T cells, a pattern consistent with mechanisms proposed in ICI-associated vasculitis (7). ICIs may elicit vasculitis *de novo* or precipitate flares of pre-existing disease (8).

For the diagnostic evaluation of these conditions, a thorough history and physical examination are essential. The laboratory work-up should include inflammatory markers, viral serologies, cryoglobulins, and urinalysis, with testing for ANA, ANCA, and complement levels. Contrast-enhanced CT remains the modality of choice due to its high sensitivity and specificity, typically revealing concentric mural thickening of the affected vessel wall, while 18F-FDG PET/CT demonstrates graded inflammatory uptake.

In this anatomic setting, we did not pursue arterial wall biopsy because in ICI-associated large-vessel vasculitis, the diagnostic yield is low while procedural risks (hemorrhage, dissection, neurovascular injury) are nontrivial. Contemporary guidelines favor noninvasive imaging as first-line confirmation, reserving biopsy for superficial arteries when cranial giant cell arteritis is suspected (9, 10). With isolated aortic or supra-aortic involvement, deep arterial biopsy is seldom undertaken outside surgical contexts and rarely alters immediate management, which is driven by clinicoradiologic concordance.

From a management standpoint, ICI therapy should be withheld and prednisone (or equivalent) should be initiated at 0.5–2 mg/kg/day according to severity, with a gradual taper over 4–6 weeks. If there is no clinical response within 48–72 hours, the addition of an immunosuppressant (tocilizumab, rituximab, or infliximab) is recommended (11).

The prognosis of patients with ICI-associated vasculitis is generally favorable, with high rates of clinical response after withholding the ICI and initiating systemic glucocorticoids. With respect to the continuation of cancer therapy, standard practice is to discontinue the ICI temporarily or permanently, particularly in the presence of significant organ involvement. Nevertheless, in selected cases and after resolution of the adverse event, rechallenge has been reported with a low risk of relapse. In an international cohort, only 1 of 9 re-exposed patients experienced recurrence (12). Decisions to reinitiate ICIs should be individualized, considering the severity of vasculitis and the status of tumor control (11). In this *KRAS-G12C*–mutated adenocarcinoma, transitioning to adagrasib yielded a durable partial response and acceptable tolerability, illustrating that effective non-ICI options can preserve oncologic control when severe irAEs preclude immunotherapy.

Immune checkpoint inhibitor–associated aortitis entails a persistent risk of serious structural complications (most notably dissection or aneurysm) even after clinical remission and normalization of inflammatory markers. Because inflammatory activity may precede or coexist with structural damage, long-term, imaging-led surveillance is warranted. Serial contrast-enhanced CT angiography or magnetic resonance angiography should be used to track aortic diameter, wall abnormalities, and branch involvement. 18F-FDG PET/CT is reserved for assessing inflammatory activity in acute or relapse settings and does not replace structural follow-up. The choice between CT angiography and magnetic resonance angiography should be individualized according to renal function, contrast allergies, radiation considerations, age, and local expertise. A practical schedule (extrapolated from large-vessel vasculitis and aortopathy guidance) is imaging at 1, 6, and 12 months (13), then annually if stable, with intervals adjusted to 6–24 months based on disease activity, vascular extent, and clinical stability. Intervals should be shortened if aneurysmal growth accelerates, new symptoms arise, or complications are detected. Management should be multidisciplinary (rheumatology, cardiology, radiology) with early input from vascular/cardiothoracic surgery when surgical thresholds are approached. In our patient, multidisciplinary Heart Team discussion was pivotal: given the asymptomatic dissection and advanced malignancy, the risks of elective repair were deemed to outweigh its potential benefits, supporting a conservative strategy with optimal medical therapy and close imaging surveillance. This underscores the central role of multidisciplinary committees in complex cardio-oncology decision-making.

## 5 Conclusion

This case adds to the limited evidence base by characterizing an unexpected pattern of findings: persistent fever with negative infectious studies and normal procalcitonin, cross-sectional and metabolic imaging consistent with aortitis, brisk steroid responsiveness with complete metabolic normalization on follow-up PET/CT and, notably, a subsequent incidental Stanford type A aortic dissection months later despite prior metabolic quiescence. Together, these features underscore the need to retain vasculitis in the differential diagnosis of fever during ICI therapy and to implement structured vascular surveillance when the aorta is involved.

## Data Availability

The original contributions presented in the study are included in the article/**Supplementary Material**. Further inquiries can be directed to the corresponding author.
